# Trace Amounts of Co_3_O_4_ Nano-Particles Modified TiO_2_ Nanorod Arrays for Boosted Photoelectrocatalytic Removal of Organic Pollutants in Water

**DOI:** 10.3390/nano11010214

**Published:** 2021-01-15

**Authors:** Yongling Du, Zhixiang Zheng, Wenzhuo Chang, Chunyan Liu, Zhiyong Bai, Xinyin Zhao, Chunming Wang

**Affiliations:** 1 College of Chemistry and Chemical Engineering, Lanzhou University, Lanzhou 730000, China; liuchy19@lzu.edu.cn (C.L.); baizhy17@lzu.edu.cn (Z.B.); Wangcm@lzu.edu.cn (C.W.); 2 Key Laboratory of Evidence Science Techniques Research and Application, Gansu University of Political Science and Law, Lanzhou 730070, China; zhaoxy18409492375@163.com; 3 College of Chemistry and Chemical Engineering, Northwest Normal University, Lanzhou 730000, China; 15800951150@163.com

**Keywords:** photocatalytic degradation, photo-electrocatalysis, Co_3_O_4_-TiO_2_, dyes, organic pollutants

## Abstract

Trace amounts of Co_3_O_4_ modified TiO_2_ nanorod arrays were successfully fabricated through the photochemical deposition method without adding any nocuous reagents. The Co_3_O_4_/TiO_2_ nanorod arrays fabricated in acid solution had the highest photo-electrochemical activity. We elaborated on the mechanism of Co_3_O_4_-TiO_2_ fabricated in different pH value solutions. The Co_3_O_4_-TiO_2_ had a more remarkable photo-electrochemical performance than the pure TiO_2_ nanorod arrays owing to the heterojunction between Co_3_O_4_ and TiO_2_. The degradation of methylene blue and hydroquinone was selected as the model reactions to evaluate the photo-electrochemical performance of Co_3_O_4_-TiO_2_ nanorod arrays. The Co_3_O_4_/TiO_2_ nanorod arrays had great potential in waste water treatment.

## 1. Introduction

From the comprehensive point of view, semiconductor metal oxides have been widely used as stable photo-catalysts for the cosmopolitan energy crisis [1,2,3,4,5]. TiO_2_ is the most extensively used semiconductor photo-catalyst owing to its exceptional properties, such as high photo-catalytic activity, chemical stability, environmental-friendliness, and low cost [3,4,6,7,8]. However, because of the large bandgap of TiO_2_ (3.2 eV), the practical applications are hampered by its low electrical conductivity, strong reflection, and weak light-harvesting ability [9,10], as well as the rapid combination of photo-generated electron and hole pairs. Various strategies have been utilized to improve the photo-catalytic efficiency of TiO_2_ materials, such as tuning their crystallite size and structure, sensitizing them by organic dye and quantum dots [8,11,12], and modifying them with metals (e.g., Pt, Ru, Ag, Au, Rh, Pd, Ni, and Co) [3,10,13,14,15,16,17] or transition metal oxides (e.g., Co_3_O_4_, CoO, Cu_2_O, and Fe_2_O_3_) [18,19,20,21] with a narrow band gap semiconductor. The formation of hetero-junctions between metal oxides and semiconductors is a useful strategy to suppress the recombination of photo-generated electrons and holes in TiO_2_, and extend photon absorption into the visible regime, which can enhance the photochemical efficiency of TiO_2_ nanomaterials.

Semiconductor-based hetero-junctions are able to facilitate fast charge separation and enhance the photo-catalytic efficiency of TiO_2_ nanomaterials. It is an effective strategy to construct TiO_2_-based hetero-junction structures with transition metal oxides. Heterojunction structures have the potential to facilitate electron–hole separation. TiO_2_ is a n-type semiconductor and, combined with a p-type semiconductor in a suitable band gap position to form a p-n heterojunction, it is an effective tactic to expand light absorption, enhance the separation effects of electrons and holes, prolong the lifetime of the electron and hole, and heighten the photocatalytic activity. Transition metal oxides, such as Ag_2_O, Cu_2_O, CuO, and Co_3_O_4_, have been used to form a p-n junction to promote an interfacial electron transfer process and increase the separation effect [18,22,23,24]. Cobalt oxides have received attention because of their excellent photo-catalytic activity in carbon dioxide reduction, oxygen reduction, and environment restoration [25,26]. Based on its properties of outstanding photocatalytic activity and low cost, cobalt oxide becomes a feasible material to fabricate the heterojunction structure with other semiconductor photocatalysts. In this work, the Co_3_O_4_-TiO_2_ nanorod arrays were synthesized by photochemical deposition (a green method). The performance of Co_3_O_4_-TiO_2_ nanorod arrays could be controlled by adjusting the pH value and the concentration of the Co precursor.

## 2. Experimental

### 2.1. Preparation of TiO_2_ Nanorod Arrays

The TiO_2_ nanorod arrays on Fluorine doped tin oxide (FTO) were fabricated through the modified hydrothermal method [7,8]. Typically, 30 mL HCl (6 mol/L) was mixed with 0.4 mL tertrabutyl titanium by strong stirring for 10 min. Then, the above solution was transferred into a Teflon pot, in which an FTO glass electrode with the coated layer facing down was placed against the wall of the Teflon pot. The hydrothermal synthesis was carried out at 150 °C for 6 h in an oven. Then, the TiO_2_ nanorod arrays was washed with high pure water, and dried by high pure N_2_.

### 2.2. Fabrication of Ultra Small Co_3_O_4_ Coated TiO_2_ Nanorod Arrays

The Co_3_O_4_-coated TiO_2_ nanorod arrays were prepared by photo-chemical deposition in a 1:1 ethanol/water solution containing different concentrations of Co(NO_3_)_2_ under Xe lamp with a powder intensity of 100 mW/cm^2^. The driving force of the Co deposition on the TiO_2_ nanorod arrays was high energetic photons, which could photo-excite electrons from the valance band of TiO_2_ to the conductor band and leave holes on the valance band. The holes were depleted by the hole receptor of ethanol. The electrons can reduce the cobalt ions onto the surface of TiO_2_ nanorod arrays. Nano cobalt metal was stable and easily oxidized to Co_3_O_4_.

### 2.3. Photo-Electrochemical Studies of the Co_3_O_4_ Modified TiO_2_ Nanorod Arrays

To study the photo-electrochemical response of the Co_3_O_4_-modified TiO_2_ nanorods and pure TiO_2_ nanorod arrays (1.0 cm × 1.0 cm), open circuit potential (OCP) and the ampere-metric method (*I*–*T*) were conducted in 0.1 mol/L Na_2_SO_4_ solution at room temperature after being deoxidized by high pure N_2_ for 15 min.

### 2.4. The Photo-Electrochemical Degradation Research

Methylene blue and hydroquinone were used to test the photo-electrochemical activity of Co_3_O_4_ modified TiO_2_ nanorod arrays and pure TiO_2_ nanorod arrays. The Co_3_O_4_ modified TiO_2_ and pure TiO_2_ nanorod arrays with a geometric area of 1.0 cm × 1.0 cm were used as the working electrode, as well as platinum as the counter electrode and saturated calomel electrode (SCE) as the reference electrode. A 500 W Xe lamp was used to simulate sunlight with a powder intensity of 100 mW/cm^2^. A solution containing 10 mg/L methylene blue or hydroquinone, 0.1 mol/L Na_2_SO_4_, and 10 mmol/L H_2_O_2_ was used as the investigated subject. During the photo-electrochemical degradation process, the electrode was added with 1.0 V bias potential (*vs*. SCE) and was light-illuminated to degrade methylene blue or hydroquinone. To avoid the effects of solution temperature, the reaction system was placed in a constant temperature system with a circulating water device (Beijing LabTech Instruments Co., Ltd., Beijing, China). The photo-electrochemical degradation process of methylene blue and hydroquinone was measured by UV/vis spectrum.

## 3. Results and Discussions

### 3.1. XRD Analysis

**Figure 1:**
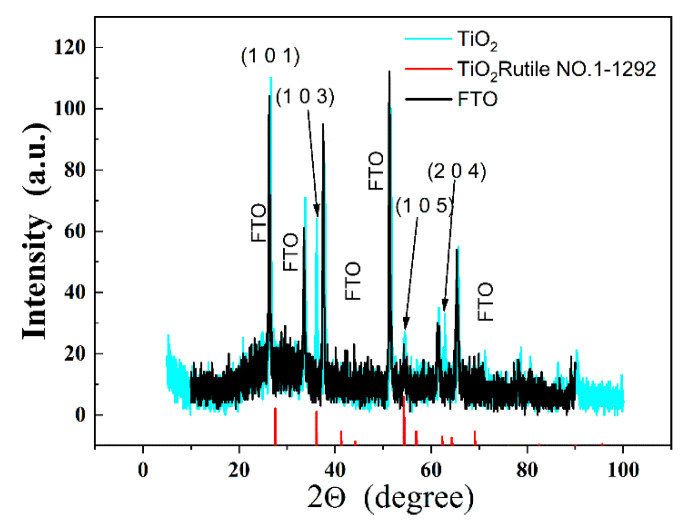
XRD pattern of Co_3_O_4_-TiO_2_ nanorod arrays and pure TiO_2_ nanorod arrays.

The Co_3_O_4_-TiO_2_ and TiO_2_ nanorod arrays were investigated by X-ray diffraction measurement (XRD) using copper target to identify the phase of all samples. The Co_3_O_4_-TiO_2_ and TiO_2_ samples in Figure 1 show the rutile phase of TiO_2_ (JCPDS No1-1292). No new XRD peaks of Co_3_O_4_ were observed for the Co_3_O_4_-TiO_2_ nanorod arrays, which might because of the small amount of cobalt oxide in the Co_3_O_4_-TiO_2_ hybrid catalyst.

### 3.2. Scanning Electron Microscopy (SEM) and Transmission Electron Microscopy (TEM) Measurement

**Figure 2:**
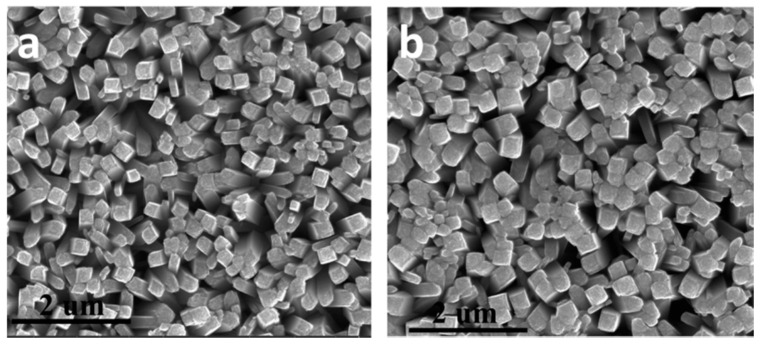
Scanning electron microscopy (SEM) images of (**a**) TiO_2_ nanorod arrays and (**b**) Co_3_O_4_-TiO_2_ nanorod arrays.

The morphologies and microstructures of the TiO_2_ nanorod arrays and Co_3_O_4_-TiO_2_ nanorod arrays were characterized by scanning electron microscopy (SEM). Figure 2 shows the top view SEM images of the TiO_2_ and Co_3_O_4_-TiO_2_ nanorod arrays. Highly ordered and large scale TiO_2_ nanorod arrays are vertically aligned on both pure TiO_2_ and Co_3_O_4_-TiO_2_ nanorod arrays in Figure 2.

The average diameter of TiO_2_ nanorod is about 180 nm with a rectangular cross section. There are almost no different features between Co_3_O_4_-TiO_2_ nanorod arrays and pure TiO_2_ nanorod arrays, which might because of the small amount of Co element in the Co_3_O_4_-TiO_2_ nanorod arrays. The small amount of Co element could be confirmed by Energy Dispersive X-ray (EDX) in Supplementary Materials Figure S1. Compared with the super strong signal of Ti and O element, the signal of Co element was very low, which means the cobalt content was very low.

**Figure 3:**
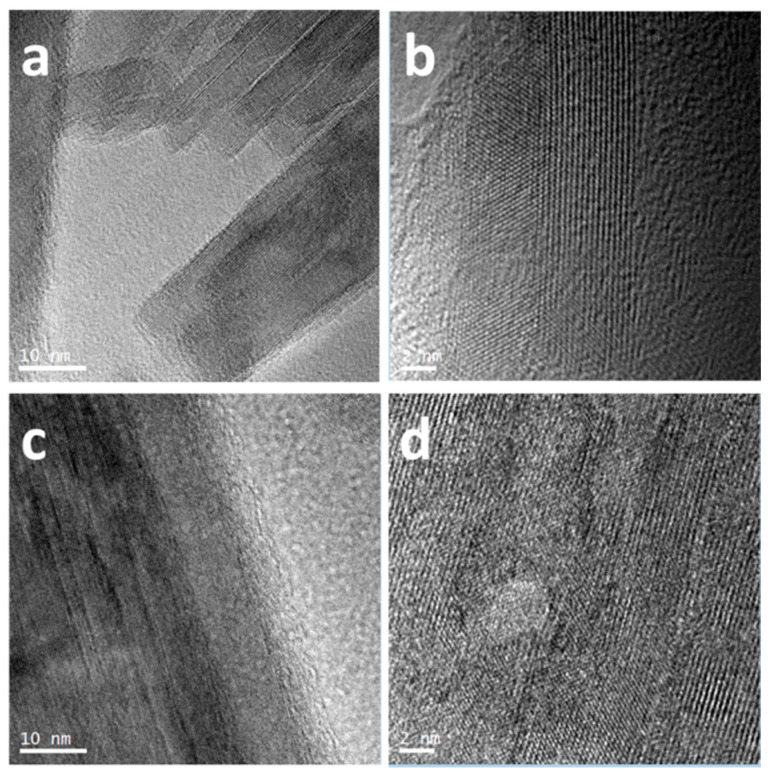
TEM and high resolution TEM of TiO_2_ nanorod arrays (**a**,**b**) and Co_3_O_4_-TiO_2_ nanorod arrays (**c**,**d**).

Figure 3a–d show the TEM images of TiO_2_ nanorods and Co_3_O_4_-TiO_2_ nanorod arrays, respectively. It can be clearly seen that the TiO_2_ nanorod arrays have the dimension of 4 nm with a clearly lattice structure. Figure 3b is the high resolution of TEM image of TiO_2_. Compared with the clear lattice structure of pure TiO_2_ nanorods, the surface of Co_3_O_4_-TiO_2_ nanorod arrays (Figure 3c) was covered with something similar to fog, which makes the lattice structure of Co_3_O_4_-TiO_2_ nanorods not obvious. High resolution TEM of Co_3_O_4_-TiO_2_ nanorod in Figure 3d showed that there were some amorphous Co_3_O_4_ on the surface of TiO_2_.

### 3.3. X-ray Photoelectron Spectroscopy (XPS) Analysis

**Figure 4:**
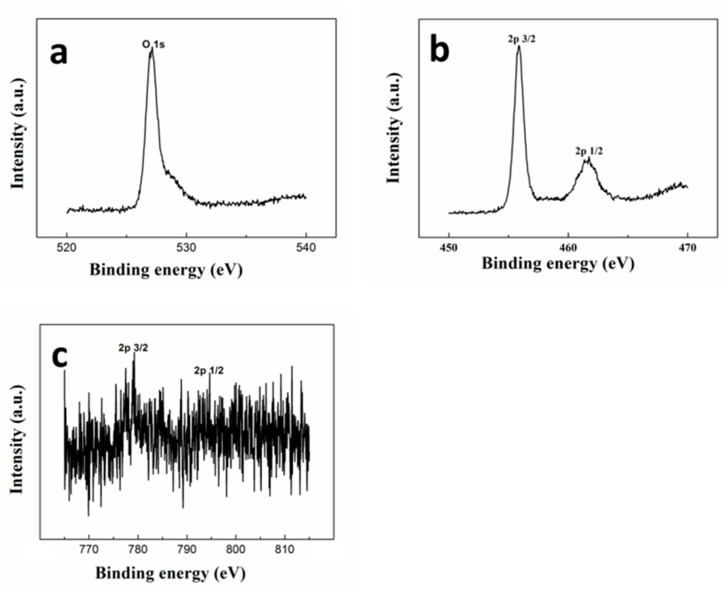
The XPS spectra of Co_3_O_4_-TiO_2_ nanorod arrays: (**a**) O _1s_, (**b**) Ti_3d_, and (**c**) Co_2p_.

To confirm the composition of Co_3_O_4_-TiO_2_ nanorod arrays sample, the XPS method was used to study the chemical composition and valence state of Co_3_O_4_-TiO_2_. The survey spectra illustrated in Supplementary Materials Figure S2 demonstrates the existence of Co, Ti, and O elements. Figure 4a shows the XPS spectrum of O _1s_. Figure 4b shows the XPS spectrum of Ti 2p obital of the Co_3_O_4_-TiO_2_ nanorod array. The Ti 2p_3/2_ and 2p_1/2_ located at 458.4 eV and 464.1 eV can be assigned to Ti^4+^, which coincided with TiO_2_. The band energy of 780.50 eV and 797.43 eV in Figure 4c corresponded to Co 2p_3/2_ and Co 2p_1/2_, respectively. The peaks are the typical signature of Co_3_O_4_ and are consistent with the previous literature [27]. Compared with the strong intensity of the Ti and O element, the XPS spectra strength of cobalt was very weak, which meant the amount of cobalt element in Co_3_O_4_-TiO_2_ nano-materials was small.

The experimental data were in accordance with the EDX results in Supplementary Materials Table S1. The amount of Co was small, because of the high activity of cobalt metal. Cobalt element is more vivacious than hydrogen element, which means there is a competitive reaction between cobalt ions and hydrogen ions during photochemical deposition. The competition between cobalt ions and hydrogen ions decreases the amount of cobalt deposition on the TiO_2_ surface. Furthermore, the Co metal easily dissolved into Co ions under acid circumstance. Therefore, the competitive reaction and the instability of cobalt lead to the ultra-small amount of Co on the TiO_2_ nanorod arrays.

### 3.4. Photocurrent Test

**Figure 5:**
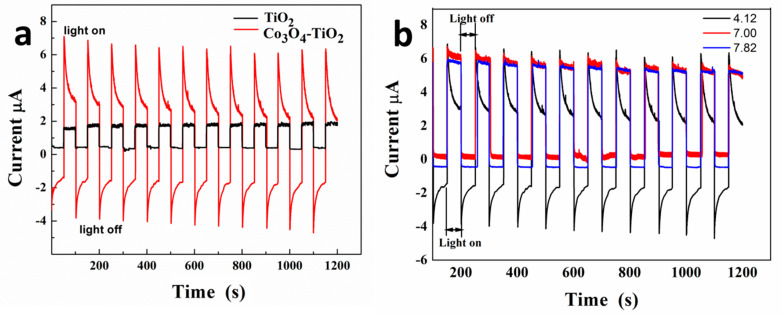
The *I*–*T* curves measured at applied potential of 0 V *vs.* saturated calomel electrode (SCE) under chopped illumination in 0.1 mol/L Na_2_SO_4_ solution: (**a**) TiO_2_ and Co_3_O_4_-TiO_2_ fabricated at pH 4.12; (**b**) Co_3_O_4_-TiO_2_ fabricated at different pH value.

The transient photocurrent was further used to confirm the generation, transfer, and separation processes of the photo-induced electrons and holes on both Co_3_O_4_ modified TiO_2_ nanorod arrays and the pure TiO_2_ nanorod arrays. To illustrate the effect of Co_3_O_4_ on photocatalytic activity of Co_3_O_4_-TiO_2_ nanorod array, the photocurrent response of the TiO_2_ nanorod arrays and Co_3_O_4_-TiO_2_ nanorod arrays was measured by chopping light. The curves of both Co_3_O_4_-TiO_2_ and TiO_2_ samples in Figure 5a had outstanding responses to chopping light cycles. The current value of Co_3_O_4_-TiO_2_ is five times higher than pure TiO_2_ nanorod arrays. This means that the Co_3_O_4_-TiO_2_ nanorod arrays had higher photoelectrocatalytic activity than the pure TiO2 nanorod arrays.

Compared with pure TiO_2_ nanorod arrays, the Co_3_O_4_-TiO_2_ nanorod arrays exhibited an obviously higher photocurrent, which indicated that Co_3_O_4_-TiO_2_ nanorod arrays had the higher photo-electrochemical activity. Under light illumination, the electron in the valence band was excited to the conductor band and left a hole in the valence band. The electrons were accumulated on the conductor band and holes were assembled on the valence band by persistent light illumination [28]. The relative OCP value of the samples was measured to compare the performance in a different semiconductor. In comparison, the relative OCP value of Co_3_O_4_-TiO_2_ was higher than the pure TiO_2_ nanorod arrays, which demonstrated that separation of e-h^+^ pairs in the Co_3_O_4_-TiO_2_ heterojunction is significantly improved by the addition of Co_3_O_4_ (Supplementary Materials Figure S3).

### 3.5. The Formation Mechanisms of Co_3_O_4_ Nanoparticles

Further observation found that Co_3_O_4_-TiO_2_ nanorods fabricated in different pH values had diverse responses to visible light, as shown in Figure 5b. The Co_3_O_4_-TiO_2_ nanorods fabricated under pH 4.12 had the highest photo-electrochemical response compared with others fabricated at pH 7.82 and 7.00. The formation mechanisms of Co_3_O_4_ nanoparticles in different pH solutions were different, which led to a different response to visible light. In neutral solution, the Co_3_O_4_ were formed under photochemical deposition.

(1) $$3\mathrm{Co}-{8e}^{-}{+4H}_{2}{O=\mathrm{Co}}_{3}O_{4}{+8H}^{+}$$

Co nanoparticles were easily deposited onto the semiconductor in alkaline solution. Such as Co-ZnO was fabricated, which had high catalytic activity in oxygen production [29]. And the high catalytic activity of Ni-CdS nanorods was fabricated through photochemical deposition in NaOH solution containing Ni ions and methanol [30]. Holes have high energy, which could oxidize methanol adsorbing on the semiconductor. Considering the high concentration of ethanol, ethanol was oxidized to formaldhyde and holes were decomposed. It is very difficult to deposit transition metal or metal oxide onto semiconductors in the acid solution because cobalt metal is more active than the hydron element. There were two different competitive reactions during the photodeposition reaction. They are listed below:

(2) $${\mathrm{Co}}^{2+}{+2e}^{-}=\mathrm{Co}$$

(3) $$2H^{+}+{2e}^{-}=H_{2}$$

Furthermore, the high activity of transition metals meant they easily dissolved in acid solution. The Co metal was not stable because of oxidization reaction and photocorrosion. Considering the above reasons, the amount of Co is very small.

(4) $$\mathrm{Co}-2e^{-}={\mathrm{Co}}^{2+}$$

(5) $$3\mathrm{Co}-8e^{-}+4{\mathrm{OH}}^{-}={\mathrm{Co}}_{3}O_{4}+{4H}^{+}$$

In alkaline solution, the Co ions mainly existed through Co(OH)_4_^2−^, which was reduced to Co metal by electrons photo-excited on the TiO_2_ surface. The Co metal was not stable and could easily be oxidized to Co_3_O_4_ by oxygen or OH radical produced in photochemical deposition.

(6) $$\mathrm{Co}{\left(\mathrm{OH}\right)}_{4}{}^{2-}+{2e}^{-}={\mathrm{Co}+4\mathrm{OH}}^{-}$$

**Figure 6:**
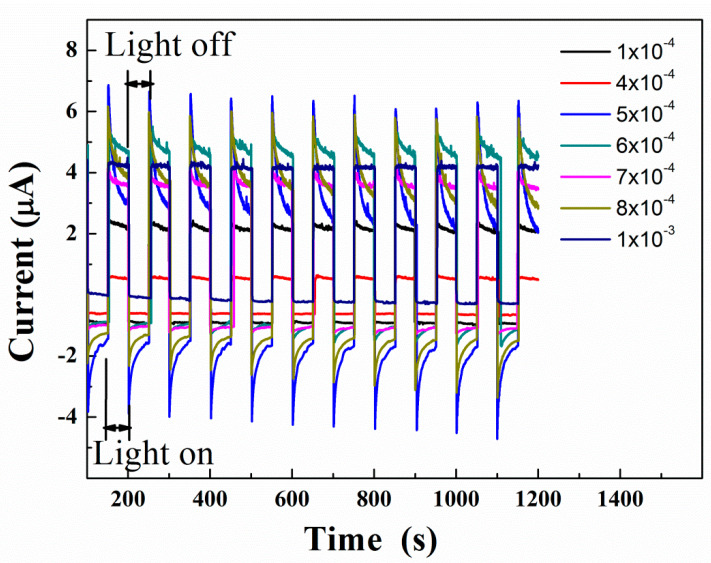
Open circuit potential (OCP) curve of Co_3_O-TiO_2_ fabricated in different concentrations of Co ions.

The amount of Co_3_O_4_ can be controlled through regulating the concentration of Co ions from 0.1 mmol/L to 1 mmol/L. We found that Co_3_O_4_-TiO_2_ nanorod array fabricated in 0.5 mmol/L cobalt ions had the highest photocatalytic activity. It can be clearly seen that the photocurrent increased with the concentration of Co ions increasing from 0.1 mmol/L to 0.5 mmol/L (in Figure 6). However, the photo-electrochemical currents decreased when the Co ions’ concentrations were further increased, which may be because of the formation of a thick Co_3_O_4_ layer and increase in the carrier recombination rate.

### 3.6. Photo-Electrochemical Activity of Co_3_O_4_ Modified TiO_2_ Nanorod Arrays

**Figure 7:**
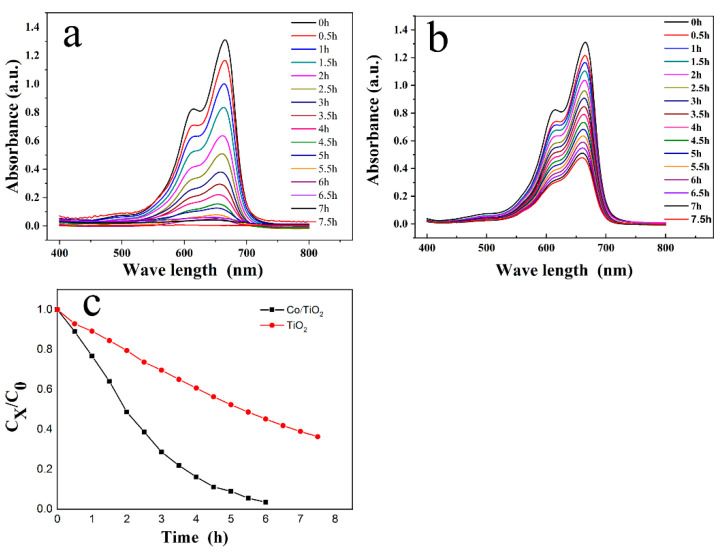
Photo-electrochemical degradation of methylene blue on Co_3_O_4_-TiO_2_ and TiO_2_ nanorod arrays: (**a**) Co_3_O_4_-TiO_2_; (**b**) TiO_2_ nanorod arrays; and (**c**) full curve of methylene blue degradation on Co_3_O_4_-TiO_2_ and pure TiO_2_.

Methylene blue was used as the probe to evaluate the photo-electrochemical activity of pure TiO_2_ nanorod arrays and Co_3_O_4_ modified TiO_2_ nanorod arrays. As shown in Figure 7, the TiO_2_ nanorod array exhibits a moderate catalytic performance for the photo-electrochemical degradation of methylene blue, which could have contributed to the ordered arrays effect. The Co_3_O_4_ modified TiO_2_ nanorod arrays exhibit excellent photo-electrochemical degradation activity to methylene blue. TiO_2_ nanorod arrays modified with Co_3_O_4_ had good photochemical catalytic activity, which was attributed to the disjunction of Co_3_O_4_-TiO_2_ and the charge transfer between Co_3_O_4_ and TiO_2_ nanometer rod components. Furthermore, the isolated Co_3_O_4_ islands on the surface of TiO_2_ nanorod arrays acted as reactive sites to enhance the photo-electrochemical activity. The degradation of methylene blue by Co_3_O_4_ modified TiO_2_ was greater than that of pure TiO_2_ nanorod arrays. Methylene blue was totally degraded on Co_3_O_4_ modified TiO_2_ nanorod arrays. Compared with Co_3_O_4_-TiO_2_ nanorod arrays, only 60% methylene blue was degraded on pure TiO_2_ nanorod arrays.

The TiO_2_ nanorod arrays only absorbed ultraviolet photons to generate e-h^+^ pairs to degrade the methylene blue molecule, while Co_3_O_4_-TiO_2_ nanorod arrays provide a p-n junction between Co_3_O_4_ nanoparticles and TiO_2_ nanorod arrays. The enhancement of photo-electrochemical activity of Co_3_O_4_/TiO_2_ heterojunction samples could have contributed to the formation of the type-II p-n hetero-junction between Co_3_O_4_ and TiO_2_. TiO_2_ is an n-type wide band gap semiconductor with the conduction band at 0.14 V, and Co_3_O_4_ is a p-type narrow band gap with band energy of 2.07 eV [31]. When Co_3_O_4_ nanoparticles are deposited onto the surface of TiO_2_ nanorod arrays, a p-n heterojunction can be formed at the surface of TiO_2_ nanorod arrays, and the electrons can be transferred from the Co_3_O_4_ to TiO_2_ nanorod arrays until their Fermi levels are equal [32]. The equilibrium can be broken by methylene blue, which acted as a holes receptor. With the photo-electrochemical reaction going on, methylene blue was degraded. The positive potential was loaded onto the Co_3_O_4_-TiO_2_ electrode to further increase the efficiency of the separation of hole and electrons. The Co_3_O_4_ has Co^2+^ and Co^3+^ valence state. Co^2+^ and Co^3+^ can be easily oxidized to Co^4+^, and Co^4+^/Co^3+^ had high activity to oxidize methylene blue to water and carbon dioxide. Thus, Co_3_O_4_-TiO_2_ had higher photo-electrochemical activity than pure TiO_2_ nanorod arrays.

**Figure 8:**
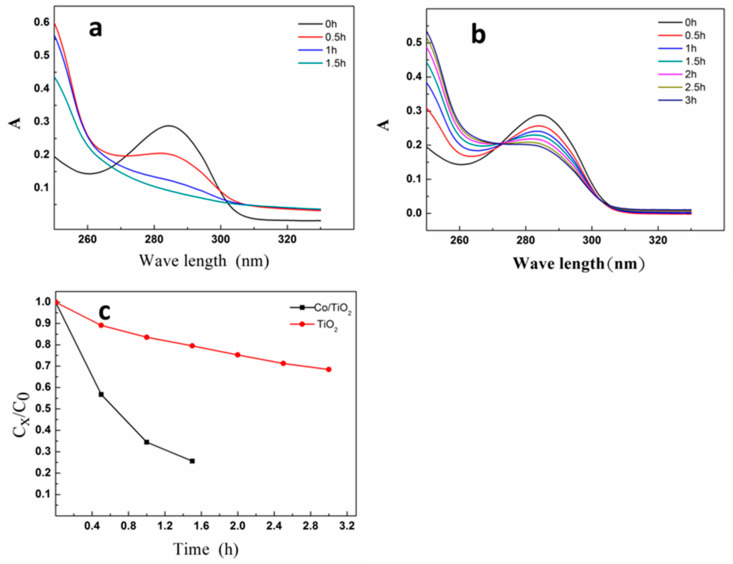
(**a**) Hydroquinone degradation on Co_3_O_4_-TiO_2_ nanorod arrays; (**b**) hydroquinone degradation on pure TiO_2_ nanorod arrays; and (**c**) comparison of degradation rate of hydroquinone on Co_3_O_4_-TiO_2_ nanorod arrays and pure TiO_2_ nanorod arrays.

Hydroquinone was used to further study the photo-electrochemical activity of Co_3_O_4_ modified TiO_2_ and pure TiO_2_ nanorod arrays. It can be seen clearly from Figure 8a,b that, compared with pure TiO_2_, the Co_3_O_4_ modified TiO_2_ nanorod arrays had higher photo-electrochemical activity. Only 30% hydroquinone was degraded on pure TiO_2_ nanorod arrays, and hydroquinone displayed total degradation on Co_3_O_4_ modified TiO_2_ nanorod arrays.

In order to clearly measure the photo-electrochemical activity of Co_3_O_4_ modified TiO_2_ nanorod arrays and pure TiO_2_ nanorod arrays, the corresponding kinetic constant was computed through fitting the experimental degradation of hydroquinone by the following equation.

(7) $$-\ln\left(\frac{C_{t}}{C_{0}}\right)=kt$$

where *C_t_* is the concentration of hydroquinone at a certain reaction time, *C*_0_ is the original concentration, *k* is the apparent first rate, and *t* is photo-electrochemical time. The model was suitable for the photo-electrochemical hydroquinone degradation process. The *k*-values for the Co_3_O_4_-TiO_2_ and pure TiO_2_ nanorod arrays are 0.91745 and 0.1206, respectively. The *k*-value of Co_3_O_4_-TiO_2_ is almost eight times that of the pure TiO_2_ nanorod arrays, which further confirmed that Co_3_O_4_ addition greatly enhanced the photo-electrochemical activity.

### 3.7. Photo-Electro-Catalytic Degradation Mechanism

**Figure 9:**
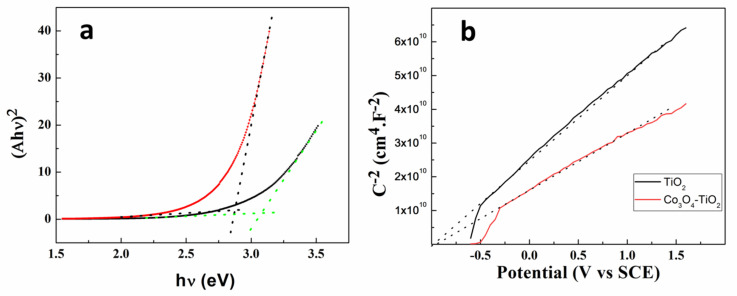
(**a**) Plots of (ahv)^2^ *vs*. photo energy of Co_3_O_4_-TiO_2_ and TiO_2_ nanorod arrays. (**b**) Mott–Schottky plots of Co_3_O_4_-TiO_2_ and pure TiO_2_ nanorod arrays in 0.5 mol/L Na_2_SO_4_ at a frequency of 1 KHz.

The photo-electrochemical activity of the photo-catalyst was mainly determined by light absorption, charges holes separation, and charge transfer from the inner to the surface of catalyzer. The band gap of the semiconductor was the key factor, which had a great influence on the photo-activity of the catalyst. The bandgap of Co_3_O_4_-TiO_2_ nanorod arrays and pure TiO_2_ nanorod arrays was determined from UV/vis diffuse reflectance spectra using the Tauc function, as shown in Figure 9a. The bandgap of TiO_2_ nanorod arrays is 3.2 eV and is very close to that from the former literature [33]. The bandgap of Co_3_O_4_-TiO_2_ is 2.85 eV, which could have contributed to the intrinsic narrow band gap of Co_3_O_4_ hybrid with TiO_2_. The addition of Co_3_O_4_ reduced the band gap of the nanomaterial and then enhanced its catalytic activity.

The influence of Co_3_O_4_ on the energy level of the photo-catalyst was studied through the Mott–Schottky electrochemical method in 0.1 mol/L Na_2_SO_4_ with 1000 Hz for Co_3_O_4_-TiO_2_ and pure TiO_2_ nanorod arrays. Both TiO_2_ and Co_3_O_4_-TiO_2_ had positive slopes, meaning that both pure TiO_2_ and Co_3_O_4_-TiO_2_ are n-type semiconductors and electrons as the majority carriers. It can be seen that the addition of Co_3_O_4_ did not change the semiconductor type of TiO_2_, but greatly changed the Fermi level and the flat band potential of TiO_2_. This phenomenon is consistent with other reported work that the addition of cobalt would change the band energy of the nanomaterial [34]. The flat-band potential (*V*_b_) and the carrier density of nano-materials can be calculated according to the following equation:

(8) $$N_{D}=\frac{2}{e\varepsilon 0\varepsilon }\left(\frac{dE}{d\left(\frac{1}{C^{2}}\right)}\right)$$

where $e_{0}$ is the dielectronic constant of the material, ε is the permittivity of the vacuum, *e* is the element charge, N_D_ is the donor density, and *C* is the capacitance. From the slope in the plot of 1/*C*^2^ versus V in the Figure 9b, the smaller slope for the Co_3_O_4_-TiO_2_ reflects a higher electron donor density. The higher N_D_ means lower resistance, faster charge transfer, and higher electrochemical activity.

**Scheme 1:**
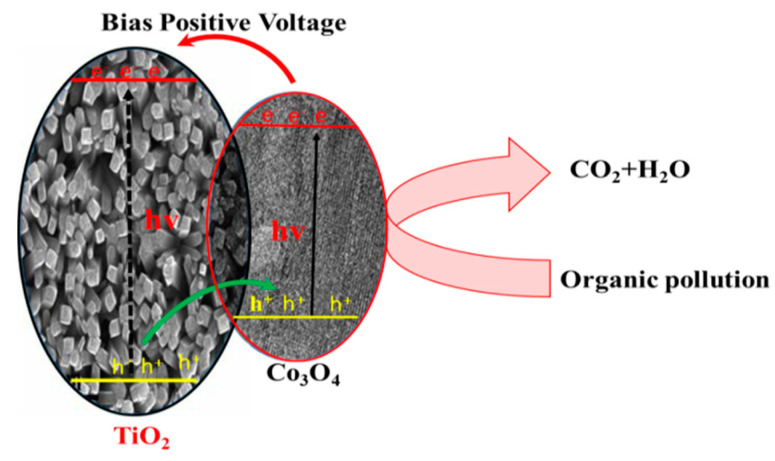
Schematic mechanism of the photo-electrochemical degradation of organic pollution on Co_3_O_4_-TiO_2_ at a constant positive bias potential.

The Co_3_O_4_-TiO_2_ nanorod arrays had greatly enhanced photo-electrochemical performance, which could be attributed to the synergetic effects of the formation of a p-n junction between Co_3_O_4_ and TiO_2_. The catalytic performance of semiconductor nanomaterials depends on the bandgap of the semiconductor nanomaterial, the separation of electrons and holes, and the lifetime of electrons and holes generated by photo exciting. The hole and electron can move the surface to react with the adsorbed reactant. However, the electron and hole could recombine easily in a short time, which greatly abates the activity of the catalyst. Therefore, the catalyst’s activity can be greatly influenced by the life-time of the photo-induced electron-holes pairs. The p-n junction could greatly enhance the life-time of the electron and holes. The longer life-time of holes and electrons greatly enhances the activity of catalysts. Co_3_O_4_ is a p-type semiconductor with a band gap of 2.19 eV [31], while TiO_2_ is an n-type semiconductor with a band gap of 3.2 eV [1]. Thus, Co_3_O_4_ participating in the TiO_2_ nanorod arrays could change the structure of TiO_2_ in three aspects: (1) broaden the absorption range from ultraviolet light to visible light; (2) form a p-n junction to enhance the life-time of the electron; or (3) the disjunct Co_3_O_4_ nanoparticles act as an activation point to improve the photo-electrochemical activity. The conduction band (CB) position of TiO_2_ is more anodic than Co_3_O_4_, so the excited electrons on the CB of TiO_2_ could not transfer to Co_3_O_4_, while the holes could transfer from TiO_2_ to Co_3_O_4_. The recombination of electron and hole could be reduced just as shown in Scheme 1. At the hetero-junction in thermal equilibrium, the p-type and n-type regions have completely opposite charges, and the n-type regions become positive, while the p-type region becomes negative. When the n-p hetero-junction semiconductor is excited by visible light with high energy to band gap, the photo-generated electrons can move to the CB of the n-type TiO_2_ and holes can move to the VB of the n-type semiconductor for the formation of the inner electric field in the Co_3_O_4_/TiO_2_ sample, which effectively impedes the recombination of electron–hole pairs. The biased voltage could further restrain the photo-excited electrons and holes recombination through the following mechanism. The positive bias voltage depletes the electrons and, as a result, the holes can be excluded to the surface. Then, the absorbed molecules on the surface of Co_3_O_4_ can react with the holes to form a series of radicals, such as OH radical and other radicals. These radicals have great energy to oxidize the organic waste to water and carbon dioxide.

(9) $${\mathrm{Co}}_{3}O_{4}+\mathrm{visible}\;\mathrm{light}\to {\mathrm{Co}}_{3}O_{4}\left(e^{-}\right)+{\mathrm{Co}}_{3}O_{4}\left(h^{+}\right)$$

(10) $${\mathrm{TiO}}_{2}+\mathrm{bias}\;\mathrm{voltage}\to {\mathrm{TiO}}_{2}\left(h^{+}\right)$$

(11) $${\mathrm{Co}}_{3}O_{4}+\mathrm{bias}\;\mathrm{voltage}\to {\mathrm{Co}}_{3}O_{4}+\mathrm{holes}$$

(12) $$H_{2}O_{2}+\mathrm{hole}\to 2\mathrm{OH}$$

(13) $$\mathrm{MB}+2\mathrm{OH}\to {\mathrm{CO}}_{2}+H_{2}O$$

According to the above results and discussion, consequently, under external bias voltage, electrons transfer along the external wires to auxiliary electrode and leave holes on the surface of Co_3_O_4_, which could oxidize the organic waste to water and carbon dioxide, as the schematic shows.

## 4. Conclusions

Ultra small amounts of Co_3_O_4_-modified TiO_2_ nanorod arrays were successfully fabricated thorough green photochemical deposition methods without adding any nocuous reagents. The Co_3_O_4_/TiO_2_ nanorod arrays fabricated in acid solution had the highest photo-electrochemical activity. We elaborated on the mechanism of Co_3_O_4_-TiO_2_ fabricated in different pH value solutions. The amount of Co_3_O_4_ could be controlled by adjusting the concentration of Co ions. The small amount of Co_3_O_4_ made many disjunct active points, which acted as active sites to mineralize organic wastes during photoelectrochemical degradation. The Co_3_O_4_/TiO_2_ nanorod arrays had higher photo-electrochemical activity to degrade organic waste than pure TiO_2_ nanorod arrays, which had great potential in waste water treatment.

## Data Availability

Data is contained within the article or Supplementary Material.

## Supplementary Materials

The following are available online at https://www.mdpi.com/2079-4991/11/1/214/s1, Figure S1: EDX of Co_3_O_4_ modified TiO_2_ nanorod arrays, Figure S2: Full XPS data of Co_3_O_4_ modified TiO_2_ nanorod arrays, Figure S3: OCP response of Co_3_O_4_ modified TiO_2_ nanorod arrays fabricated in different pH value. Table S1: the content of Co_3_O_4_ modified TiO_2_ nanorod arrays.
