# Supplementary material for: Trace Amounts of Co3O4 Nano-Particles Modified TiO2 Nanorod Arrays for Boosted Photoelectrocatalytic Removal of Organic Pollutants in Water
Source: Nanomaterials (Basel). 2021 Jan 15;11(1):214. doi: 10.3390/nano11010214 (PMC7830349; doi:10.3390/nano11010214)
Supplement: Supplementary file 1 [file nanomaterials-11-00214-s001.pdf]

Supporting Information

Trace Amounts of Co_3O_4 Nano-Particles Modified TiO_2 Nanorod Arrays for Boosted Photoelectrocatalytic Removal of Organic Pollutants in Water

Yongling Du [1,*,†], Zhixiang Zheng [2,*,†], Wenzhuo Chang [3], Chunyan Liu [1], Zhiyong Bai [1], Xinyin Zhao [2], and Chunming Wang [1]

[1] College of Chemistry and Chemical Engineering, Lanzhou University, Lanzhou 730000, China; liuchy19@lzu.edu.cn (C.L.); baizhy17@lzu.edu.cn (Z.B.); Wangcm@lzu.edu.cn (C.W.)
[2] Key Laboratory of Evidence Science Techniques Research and Application, Gansu University of Political Science and Law, Lanzhou 730070, China; zhaoxy18409492375@163.com
[3] College of Chemistry and Chemical Engineering, Northwest Normal University, Lanzhou 730000, China; 15800951150@163.com
* Correspondence: dqzhenghao@163.com (Z.Z.); duyl@lzu.edu.cn (Y.D.); Tel: +86-9318911895 (Y.D.)
† These authors contributed equally to this work.

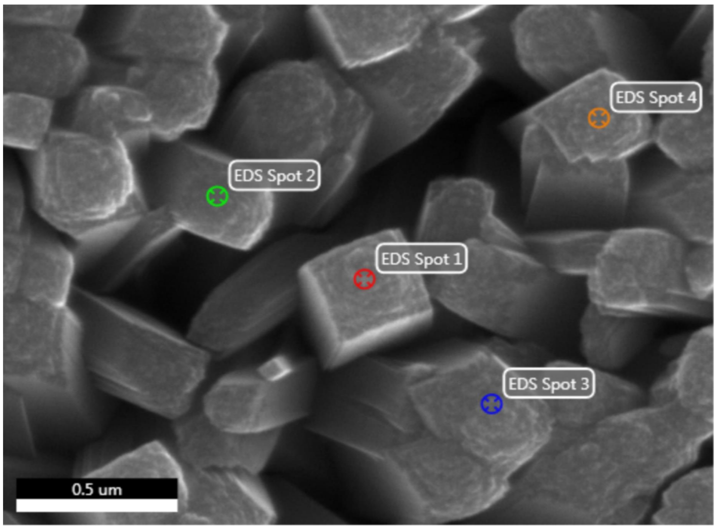

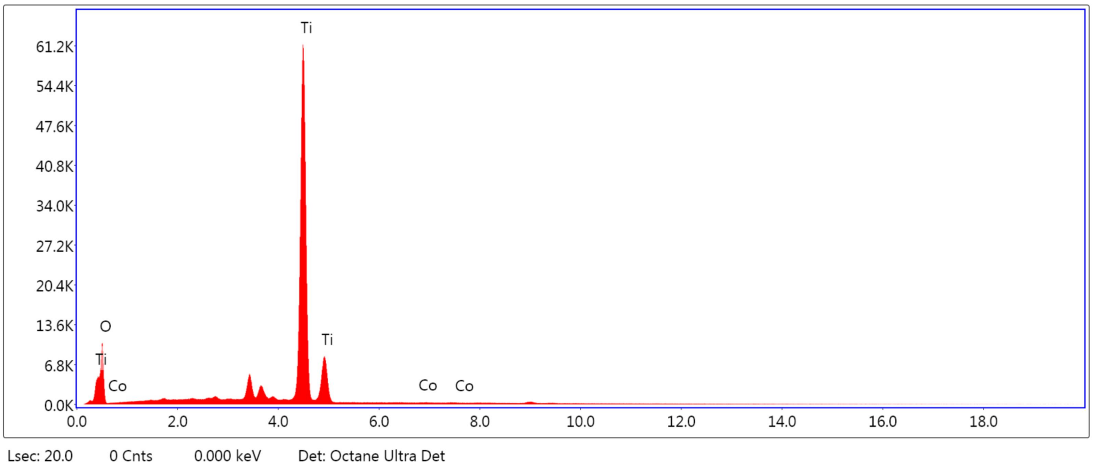

Figure S1 EDX of Co_3O_4 modified TiO_2 nanorod arrays

Table S1 the content of Co_3O_4 modified TiO_2 nanorod arrays.

Element	Weight %	Atomic %	Net Int.	Error %	Kratio	Z	R	A	F
O K	54.76	78.38	3555.82	9.97	0.0629	1.0869	0.9491	0.1055	1.0000
TiK	45.20	21.61	39524.04	1.14	0.4118	0.8759	1.0522	1.0222	1.0168
CoK	0.04	0.01	18.33	55.79	0.0003	0.8512	1.0757	0.9335	1.0587

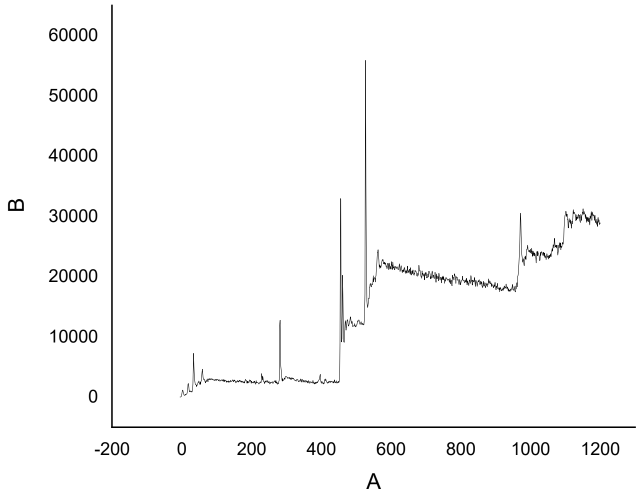

Figure S2. Full XPS data of Co_3O_4 modified TiO_2 nanorod arrays

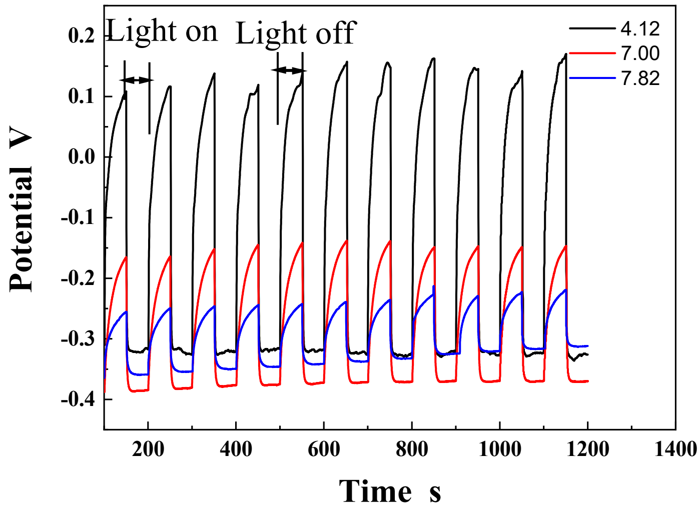

Figure S3. OCP response of Co_3O_4 modified TiO_2 nanorod arrays fabricated in different pH value.
